# Percutaneous endoscopic gastrostomy-assisted placement of endoluminal vacuum-assisted closure for esophageal perforation

**DOI:** 10.1055/a-2777-5408

**Published:** 2026-02-13

**Authors:** Ankoor H. Patel, Arvind J. Trindade, Michael Ma, Arvind Bussetty, Petros Benias

**Affiliations:** 1 12287 Rutgers Health, Robert Wood Johnson Medical School, New Brunswick, New Jersey, United States

Esophageal perforation is a rare condition with high morbidity and mortality. Management traditionally included surgical repair, primary closure with drainage, and endoscopic stenting; however, these approaches are often limited by complications such as leakage, infection, and failure of closure. Endoluminal vacuum-assisted closure (EVAC) has emerged as a minimally invasive alternative that promotes drainage and controls sepsis and facilitates healing through negative pressure therapy 1 2 . The use of EVAC presents certain challenges, including the need for multiple endoscopies and device malfunction 3 . However, the most difficult aspect is the anterograde placement of the sponge, which is a significant challenge and one of the primary reasons for the failure of placement. We present a novel technique to overcome this challenge by performing percutaneous endoscopic gastrostomy (PEG)-assisted placement of EVAC for esophageal perforation that facilitates the ease of placement and allows for enteral nutrition while the leak heals.

**Fig. 1:**
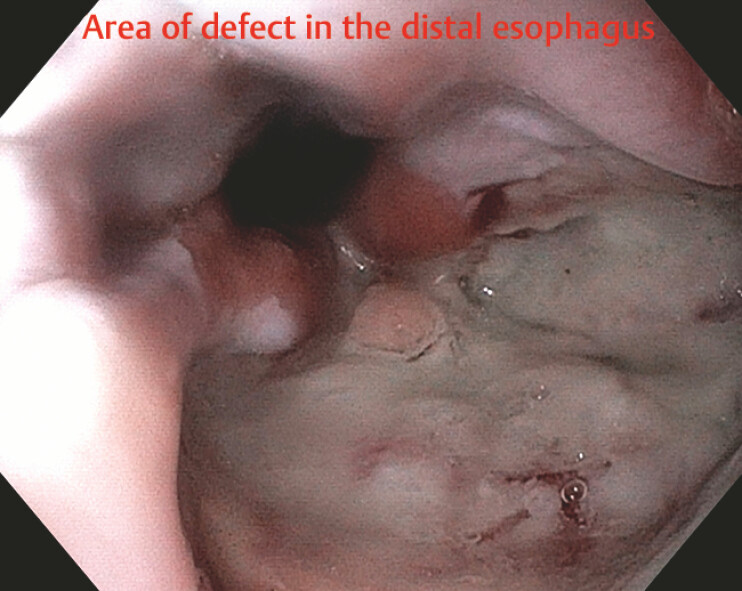
Area of an esophageal defect and after removal of a previously placed stent.

**Fig. 2:**
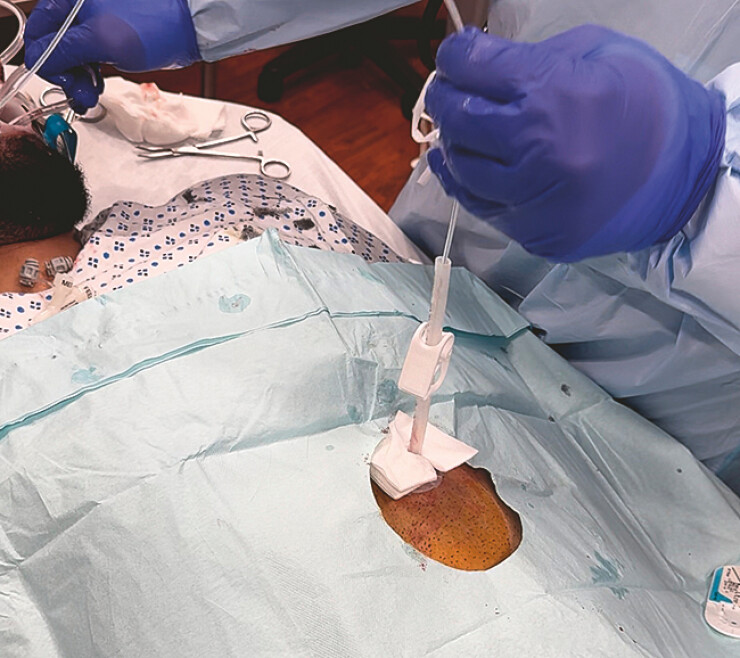
Pulling EVAC into the esophagus via snare attached to the sponge through PEG. EVAC, endoluminal vacuum-assisted closure; PEG, percutaneous endoscopic gastrostomy.

**Fig. 3:**
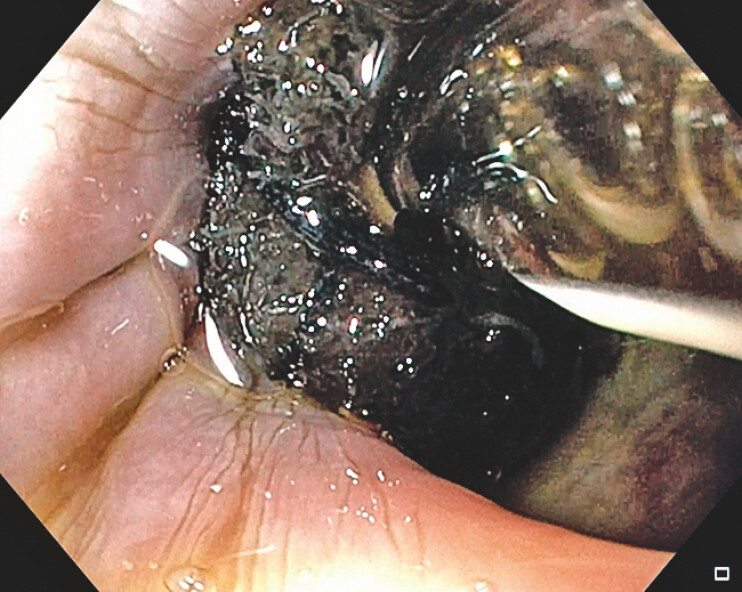
EVAC placement within the esophageal defect and at site of perforation. VAC, endoluminal vacuum-assisted closure.

We present a 30-year-old-man with chest discomfort after multiple episodes of emesis with a computed tomographic scan revealing distal esophageal perforation ( Fig. 1 ). He underwent endoscopic stent placement; however, an esophagram confirmed a persistent leak. He underwent the PEG-assisted placement of EVAC. First, a PEG tube was placed using the pull-method. The sponge was brought down to the stomach using a pull through technique using a snare which was weaved through the gastrostomy and brought up and out of the mouth using a forceps ( Video 1 , Fig. 2 ). After the sponge was brought into the stomach, we converted the PEG to a jejunal feeding tube by extending it into the distal duodenum. Finally, the sponge was pulled back until snug with the defect ( Fig. 3 ).

Percutaneous endoscopic gastrostomy-assisted placement of endoluminal vacuum-assisted closure for esophageal perforation.Video 1

This case highlights PEG-assisted EVAC placement as a novel and effective strategy in the management of esophageal perforation. By facilitating secure device placement, optimizing drainage, and establishing enteral nutrition, this technique may expend therapeutic options available for esophageal defects.

Endoscopy_UCTN_Code_TTT_1AO_2AK

Endoscopy_UCTN_Code_TTT_1AO_2AD
